# Biomarker correlation network in colorectal carcinoma by tumor anatomic location

**DOI:** 10.1186/s12859-017-1718-5

**Published:** 2017-06-17

**Authors:** Reiko Nishihara, Kimberly Glass, Kosuke Mima, Tsuyoshi Hamada, Jonathan A. Nowak, Zhi Rong Qian, Peter Kraft, Edward L. Giovannucci, Charles S. Fuchs, Andrew T. Chan, John Quackenbush, Shuji Ogino, Jukka-Pekka Onnela

**Affiliations:** 1 Program of MPE Molecular Pathological Epidemiology, Department of Pathology, Brigham and Women’s Hospital and Harvard Medical School, Boston, MA USA; 2 Department of Nutrition, Harvard T.H. Chan School of Public Health, Boston, MA USA; 3 Department of Biostatistics, Harvard T.H. Chan School of Public Health, Boston, MA USA; 4 Department of Epidemiology, Harvard T.H. Chan School of Public Health, Boston, MA USA; 5 Channing Division of Network Medicine, Department of Medicine, Brigham and Women’s Hospital and Harvard Medical School, Boston, MA USA; 6 Department of Medical Oncology, Dana-Farber Cancer Institute and Harvard Medical School, Boston, MA USA; 7 Yale Cancer Center, New Haven, CT USA; 8 Department of Medicine, Yale School of Medicine, New Haven, CT USA; 9 Smilow Cancer Hospital, New Haven, CT USA; 10 Clinical and Translational Epidemiology Unit, Massachusetts General Hospital and Harvard Medical School, Boston, MA USA; 11 Department of Biostatistics and Computational Biology, Dana-Farber Cancer Institute and Harvard Medical School, Boston, MA USA; 12 Department of Oncologic Pathology, Dana-Farber Cancer Institute and Harvard Medical School, Boston, MA USA

## Abstract

**Background:**

Colorectal carcinoma evolves through a multitude of molecular events including somatic mutations, epigenetic alterations, and aberrant protein expression, influenced by host immune reactions. One way to interrogate the complex carcinogenic process and interactions between aberrant events is to model a biomarker correlation network. Such a network analysis integrates multidimensional tumor biomarker data to identify key molecular events and pathways that are central to an underlying biological process. Due to embryological, physiological, and microbial differences, proximal and distal colorectal cancers have distinct sets of molecular pathological signatures. Given these differences, we hypothesized that a biomarker correlation network might vary by tumor location.

**Results:**

We performed network analyses of 54 biomarkers, including major mutational events, microsatellite instability (MSI), epigenetic features, protein expression status, and immune reactions using data from 1380 colorectal cancer cases: 690 cases with proximal colon cancer and 690 cases with distal colorectal cancer matched by age and sex. Edges were defined by statistically significant correlations between biomarkers using Spearman correlation analyses. We found that the proximal colon cancer network formed a denser network (total number of edges, *n* = 173) than the distal colorectal cancer network (*n* = 95) (*P* < 0.0001 in permutation tests). The value of the average clustering coefficient was 0.50 in the proximal colon cancer network and 0.30 in the distal colorectal cancer network, indicating the greater clustering tendency of the proximal colon cancer network. In particular, MSI was a key hub, highly connected with other biomarkers in proximal colon cancer, but not in distal colorectal cancer. Among patients with non-MSI-high cancer, *BRAF* mutation status emerged as a distinct marker with higher connectivity in the network of proximal colon cancer, but not in distal colorectal cancer.

**Conclusion:**

In proximal colon cancer, tumor biomarkers tended to be correlated with each other, and MSI and *BRAF* mutation functioned as key molecular characteristics during the carcinogenesis. Our findings highlight the importance of considering multiple correlated pathways for therapeutic targets especially in proximal colon cancer.

**Electronic supplementary material:**

The online version of this article (doi:10.1186/s12859-017-1718-5) contains supplementary material, which is available to authorized users.

## Background

Colorectal cancer evolves through a progressive accumulation of genetic and epigenetic alterations that are influenced by the environment and host immunity. The interplay of molecular alterations forms biological interaction networks in colorectal cancer [1]. For example, epigenetic changes are known to be associated with genetic changes, as exemplified by the well-established link between *MLH1* promoter hypermethylation and microsatellite instability (MSI) [2, 3]. Additionally, microRNAs including *MIR21* and *MIR155* are reported to regulate gene expression [4–6], resulting in abnormal loss and overexpression of proteins [7]. In the tumor microenvironment, tumor-host interactions manifest as lymphocytic reactions directed at the tumor [8]. Given this multitude of molecular interactions, it is challenging to comprehensively understand the perturbations of the complex biological system and identify the key, underlying molecular events that drive colorectal carcinogenesis. Network analysis is recognized as an emerging approach to evaluating multidimensional tumor molecular data with the aim of revealing critical molecular events and pathways [9, 10].

Numerous lines of evidence indicate differences between proximal colon cancer, and distal colon and rectal cancer [11–13]. When compared with distal colon and rectal cancer, proximal colon cancer exhibits a higher prevalence of microsatellite instability (MSI) and high-level CpG island methylator phenotype (CIMP-high) [11]. The proximal colon and distal colorectum have different embryologic origins; the former is derived from the midgut with blood supply from superior mesenteric artery, while the latter is derived from the hindgut with inferior mesenteric artery. Moreover, epithelial cells in the proximal colon are exposed to different bowel contents, including microbiota, compared with the distal colon and rectum [13]. Based on these differences in molecular alterations and tumor-host interactions with the microenvironment, the underlying etiology may differ between proximal and distal colorectal cancer. Accordingly, the structure of the biomarker correlation network may differ by tumor location. A better understanding of the connectivity and correlation of molecular events in biomarker networks can provide new insights into colorectal cancer etiology and pathogenesis, potentially advancing prevention and treatment strategies. In cancer biological networks, highly connected molecular markers, called hubs, play essential roles in carcinogenesis [14, 15]. We hypothesized that correlation network structures and hub biomarkers might differ between proximal and distal colorectal cancer. Utilizing colorectal cancer databases of the Nurses’ Health Study (NHS) and the Health Professionals Follow-up Study (HPFS), we examined the status of key driver mutations, MSI, CIMP, expression of selected proteins in tumor cells, and immune reactions to colorectal cancer.

## Results

### Comparison of biomarker networks by tumor location

Within the NHS and the HPFS, we identified 1591 colorectal cancer patients with analyzed molecular features of colorectal tumors (Table 1). Biomarkers in this analysis included somatic oncogenic mutations, epigenetic features, protein expression levels, and host immune reactions in colorectal carcinoma. We conducted network analyses stratified by tumor location in a subset (*n* = 1380) of these patients. This subset included 690 patients with proximal colon cancer and 690 patients with distal colorectal cancer, matched by age and sex. Biomarker availability differed by tumor location only for the following three biomarkers: the intratumoral periglandular reaction (95.9% available in proximal colon, 91.6% in distal colorectum), peritumoral lymphocytic reaction (95.8% in proximal colon, 91.2% in distal colorectum), and tumor infiltrating lymphocytes (TIL) (95.9% in proximal colon, 91.6% in distal colorectum), with the significance level of *P* = 0.05 after the Bonferroni correction. The demographic characteristics of patients according to tumor location are described in Table 2. Patients with proximal colon cancer tended to have tumors with higher TNM stage and poor differentiation compared with patients with distal colorectal cancer.

**Table 1:** Tumor molecular and pathological features in colorectal carcinomas

Biomarker	Status measured	% or mean (SD) of the status in proximal colon (*n* = 690)^a^	% or mean (SD) of the status in distal colorectum (*n* = 690)^a^	% availability in proximal colon (*n* = 690)	% availability in distal colorectum (*n* = 690)
Somatic oncogenic mutations					
*BRAF*	Mutation	25.1%	5.6%	86.1%	87.4%
*KRAS*	Mutation	43.0%	36.6%	86.2%	87.5%
*PIK3CA*	Mutation	18.4%	14.5%	79.7%	80.9%
Methylation status					
*MLH1*	Methylation	25.3%	3.9%	83.5%	82.5%
*CDKN2A*	Methylation	44.3%	21.8%	83.5%	82.5%
*CACNA1G*	Methylation	40.3%	9.9%	83.5%	82.3%
*CRABP1*	Methylation	52.8%	20.9%	83.5%	82.5%
*IGF2*	Methylation	44.3%	12.1%	83.5%	82.5%
*NEUROG1*	Methylation	53.1%	21.0%	83.5%	82.3%
*RUNX3*	Methylation	40.1%	8.8%	83.5%	82.5%
*SOCS1*	Methylation	30.4%	7.9%	83.3%	82.5%
LINE-1	Degree of methylation (%)	64.7 (SD, 9.7)	62.6 (SD, 9.9)	85.1%	84.5%
*IGF2* DMR0	Methylation	22.0%	29.6%	65.1%	65.2%
Microsatellite instability (MSI)					
MSI	MSI-high	28.2%	4.9%	85.9%	85.7%
Protein expression					
CDH1	Loss	52.6%	50.9%	44.9%	42.5%
CDKN1A	Loss	72.3%	89.2%	52.8%	55.2%
CDKN1B	Loss	47.9%	34.8%	51.7%	53.8%
CDKN2A	Loss	35.3%	17.9%	46.8%	47.8%
CDX2	Loss	36.6%	17.2%	43.2%	39.7%
MGMT	Loss	34.7%	38.9%	42.6%	43.9%
AURKA	Overexpression	22.2%	14.3%	32.6%	28.4%
CCND1	Overexpression	82.6%	73.5%	64.9%	65.5%
CD274	Overexpression	85.6%	83.7%	43.2%	40.0%
CDK8	Overexpression	72.6%	69.4%	30.7%	26.5%
CTNNB1 (nuclear)	Overexpression	32.7%	58.2%	75.8%	74.2%
CTSB	Overexpression	82.7%	82.9%	45.2%	42.3%
DNMT3B	Overexpression	19.7%	10.3%	45.5%	49.3%
EPAS1	Overexpression	47.0%	44.5%	46.2%	42.0%
FASN	Overexpression	61.1%	60.0%	64.8%	65.9%
HGF	Overexpression	45.8%	47.9%	39.6%	37.2%
HIF1A	Overexpression	17.0%	20.1%	47.7%	42.6%
IGF2BP3	Overexpression	36.8%	32.7%	43.8%	41.2%
IRS1	Overexpression	29.5%	30.6%	44.6%	41.7%
IRS2	Overexpression	31.6%	33.2%	44.9%	41.9%
PPARG	Overexpression	24.1%	19.2%	33.6%	29.4%
PTGER2	Overexpression	27.8%	26.3%	45.8%	41.3%
PTGS2	Overexpression	53.5%	69.7%	81.7%	79.9%
SIRT1	Overexpression	38.6%	37.9%	39.4%	35.9%
STAT3	Overexpression	54.7%	54.0%	46.4%	43.2%
TP53	Overexpression	33.0%	53.2%	66.7%	67.2%
VDR	Overexpression	37.0%	38.2%	47.4%	42.5%
YAP1 (cytoplasmic)	Overexpression	21.3%	17.7%	44.2%	41.0%
JC Virus T-Antigen (JCVT)	Overexpression	28.4%	40.7%	43.9%	47.7%
Immune reactions					
Peritumoral lymphocytic reaction	Greater reaction	19.4%	12.9%	95.8%	91.2%
Intratumoral periglandular reaction	Greater reaction	17.2%	9.0%	95.9%	91.6%
Tumor infiltrating lymphocytes (TIL)	Greater reaction	16.3%	4.4%	95.9%	91.6%
Crohn’s-like reaction	Greater reaction	9.9%	5.0%	80.4%	73.2%
CD3+ in cancer area	Density (cells/mm^2^)	716.6 (SD, 1501.8)	696.5 (SD, 1560.7)	47.1%	42.9%
CD8+ in cancer area	Density (cells/mm^2^)	814.7 (SD, 1741.0)	700.9 (SD, 1582.1)	46.7%	41.7%
CD45RO+ in cancer area	Density (cells/mm^2^)	711.4 (SD, 1113.4)	634.4 (SD, 1244.7)	48.1%	43.6%
FOXP3+ in cancer area	Density (cells/mm^2^)	40.0 (SD, 47.0)	35.2 (SD, 37.9)	45.2%	41.9%
miRNA expression					
MIR21	Normalized expression level	8.3 (SD, 13.0)	5.6 (SD, 4.0)	53.2%	48.6%
MIR155	Normalized expression level	0.01 (SD, 0.01)	0.005 (SD, 0.006)	53.2%	48.6%
Microorganism					
*Fusobacterium nucleatum*	Presence	16.0%	9.1%	71.7%	67.1%

^a^Percentage (%) indicates the proportion of the status measure for a binary biomarker, and the mean (SD) was calculated for a continuous biomarker

*SD* standard deviation

**Table 2:** Demographic, clinical and pathologic features of colorectal cancers in the network analysis dataset by tumor location

	Total (*n* = 1380)	Proximal colon cancer (*n* = 690)	Distal colorectal cancer (*n* = 690)	*P* value^a^
Age, mean (SD)	69.9 (8.6)	70.0 (8.8)	69.7 (8.5)	0.56
Sex				
Female	758 (54.9%)	379 (54.9%)	379 (54.9%)	0.99
Male	622 (45.1%)	311 (45.1%)	311 (45.1%)	
Tumor location				
Cecum	253 (18.3%)	253 (36.7%)	0 (0%)	<0.0001
Ascending colon	303 (22.0%)	303 (43.9%)	0 (0%)	
Hepatic flexure	45 (3.3%)	45 (6.5%)	0 (0%)	
Transverse colon	89 (6.4%)	89 (12.9%)	0 (0%)	
Splenic flexure	31 (2.2%)	0 (0%)	31 (4.5%)	
Descending colon	66 (4.8%)	0 (0%)	66 (9.6%)	
Sigmoid colon	296 (21.4%)	0 (0%)	296 (42.9%)	
Rectosigmoid junction	100 (7.2%)	0 (0%)	100 (14.5%)	
Rectum	197 (14.3%)	0 (0%)	197 (28.6%)	
TNM Stage				
I	324 (26.2%)	146 (22.9%)	178 (29.7%)	0.002
II	404 (32.7%)	232 (36.4%)	172 (28.7%)	
III	332 (26.8%)	159 (24.9%)	173 (28.9%)	
IV	177 (14.3%)	101 (15.8%)	76 (12.7%)	
Tumor differentiation				
Well to moderate	1231 (89.2%)	577 (83.6%)	654 (94.8%)	<0.0001
Poor	149 (10.8%)	113 (16.4%)	36 (5.2%)	
Family history of colorectal cancer in first-degree relative(s)				
No	1074 (78.4%)	532 (77.9%)	542 (78.9%)	0.65
Yes	296 (21.6%)	151 (22.1%)	145 (21.1%)	

The % numbers indicate the fraction of cases with a given feature among total cases, proximal colon cancer cases, or distal colorectal cancer cases

^a^ *P* value was calculated using a t-test for age and chi-squared tests for categorical variables

**Fig. 1:**
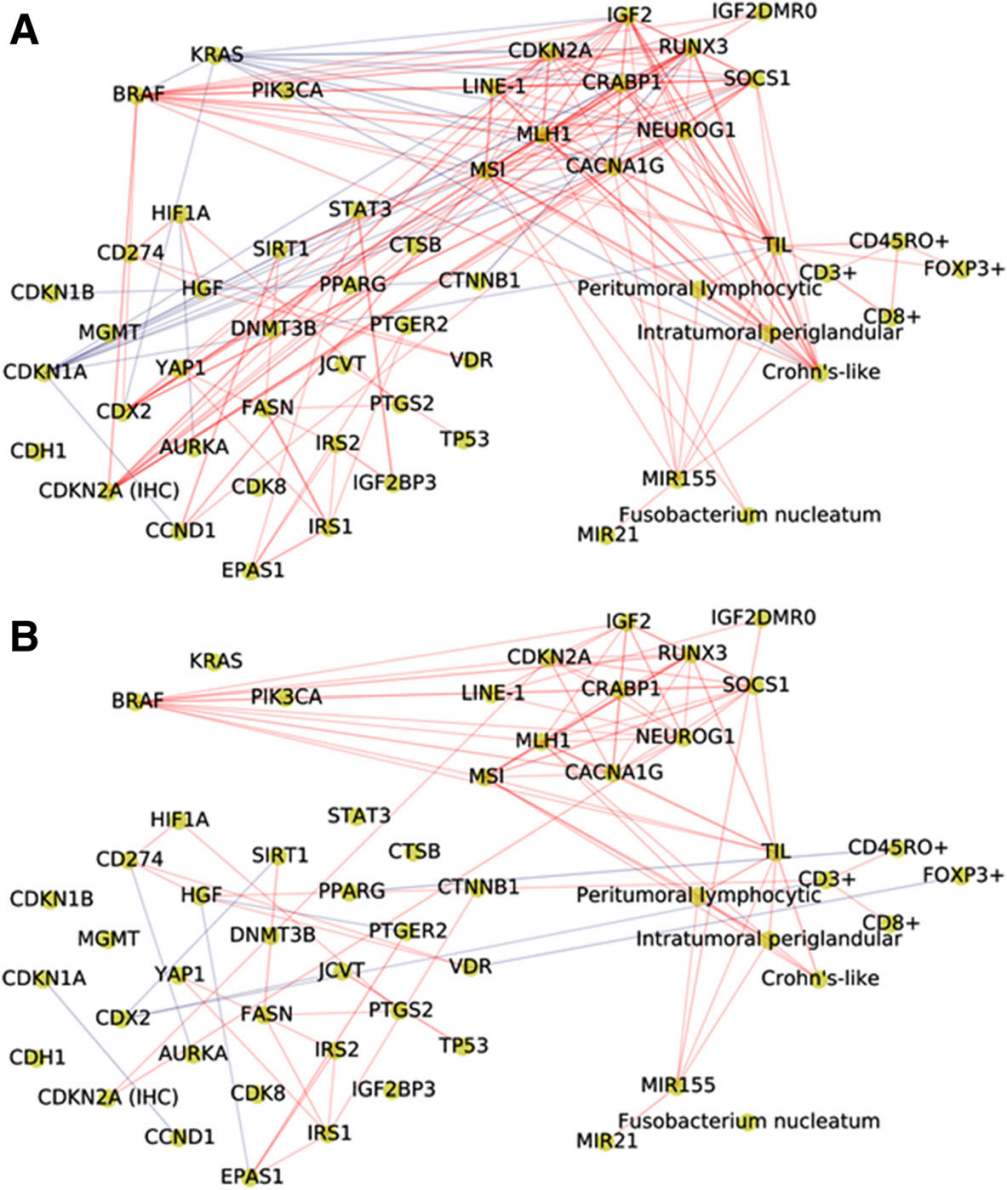
Biomarker networks by tumor location; proximal colon cancer network (**a**), and distal colorectal cancer network (**b**). A node represents a molecular feature, and an edge specifies a statistically significant Spearman correlation between two markers (nodes) with a significance level of 3.5 × 10^−5^ (0.05/1431, based on the Bonferroni correction). The red line indicates a positive correlation, and the blue line indicates a negative correlation; line width is proportional to correlation coefficient. CDKN2A (IHC), protein expression of CDKN2A; CDKN2A, methylation level of *CDKN2A*; LINE-1, methylation level of long interspersed nucleotide element 1; MSI, microsatellite instability; TIL, lymphocytes on top of neoplastic epithelial cells

**Fig. 2:**
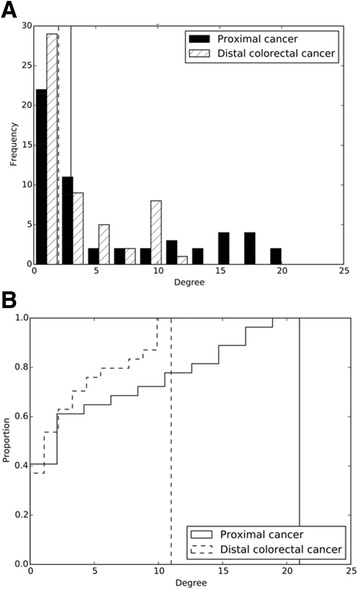
**a** Degree distribution in biomarker networks by tumor location. **b** Cumulative degree distribution in biomarker networks by tumor location. The solid (the distal colorectal cancer network) and dashed lines (the proximal colon cancer network) indicate the median degree values

In the correlation network analysis, a node represented a tumor tissue biomarker, and an edge was defined as a correlation between two nodes based on Spearman correlation analysis. Compared with the distal colorectal cancer network, the proximal colon cancer network had more edges and greater median degree (Table 3, Fig. 1). Nodes in the proximal colon cancer network tended to have a higher degree than those in the distal colorectal cancer network (*P* = 0.043 based on a K-S test, Fig. 2). The median degree value was 3.0 in the proximal colon cancer network and 2.0 in the distal colorectal cancer network. The total number of edges was significantly greater in the proximal colon cancer network (*n* = 173) than in the distal colorectal cancer network (*n* = 95) in a permutation test where tumor location variable was permuted among 1380 patients (*P* < 0.0001). Also, we observed significant difference in the total number of edges in another permutation test where values of each biomarker were permuted within proximal colon cancer patients and distal colorectal cancer patients separately (*P* < 0.0001). In addition, the value of the average clustering coefficient indicated that the nodes in the proximal colon cancer network tended to cluster together more than those in the distal colorectal cancer network (Table 3). We defined hubs as nodes with a high degree centrality (high connectivity with other nodes) based on the overall colorectal cancer network that pooled both proximal colon and distal colorectal cancer. Nodes with degree centrality greater than the 80th percentile were considered as hubs. We found hubs in the proximal colon cancer network, including MSI and *MLH1* methylation, but not in the distal colorectal cancer network (Table 3). For these biomarkers, degree centrality was computed as the fraction of nodes to which a node was connected. The values of degree centrality in the proximal colon cancer network and the distal colorectal cancer network were 0.39 and 0.17 for MSI, 0.36 and 0.21 for *MLH1* methylation. Within the distal colorectal cancer network, most of the nodes have a small number of connections. Methylation-related markers tended to have high degree centrality, although these markers were not hubs. The degree centrality values were 0.21 for *MLH1* methylation, 0.19 for methylations in *CACNA1G*, *NEUROG1*, *RUNX3*, and *SOCS1*and 0.19 for *BRAF* mutation, and 0.19 for TIL.

**Table 3:** Network characteristics by tumor location

	Total (*N* = 1380)	Proximal colon cancer (*N* = 690)	Distal colorectal cancer (*N* = 690)
Correlation network based on original variables			
Number of nodes	54	54	54
Number of edges	268	173	95
Median degree	8.0	3.0	2.0
Average clustering coefficient	0.52	0.50	0.30
Hubs^a^ (degree centrality)	*RUNX3* (0.47) *CACNA1G* (0.45) MSI (0.45) MLH1 (0.43) TIL (0.43) BRAF (0.40) *NEUROG1* (0.40) *CRABP1* (0.38) *IGF2* (0.38) *SOCS1* (0.38) *CDKN2A* ^b^ (0.36) MIR155 (0.36)	MSI (0.39) MLH1 (0.36)	-
Correlation network in non-MSI-high cancer			
Number of nodes	53	53	53
Median degree	4.0	1.0	1.0
Number of edges	120	64	56
Average clustering coefficient	0.33	0.23	0.26
Hubs^a^ (degree centrality)	*RUNX3* (0.15) *NEUROG1* (0.15)	*BRAF* (0.19) *CDKN2A* ^b^ (0.17) *IGF2* (0.17) *RUNX3* (0.17) *CACNA1G* (0.15) *CRABP1* (0.15) *NEUROG1* (0.15)	*RUNX3* (0.15) *NEUROG1* (0.15)
Correlation network based on the same number of edges			
Number of nodes	54	54	54
Median degree	1.5	4.0	1.0
Number of edges	100	100	100
Average clustering coefficient	0.27	0.29	0.27
Hubs^a^ (degree centrality)	*CACNA1G* (0.26) *IGF2* (0.26) *RUNX3* (0.25) *BRAF* (0.23) *MLH1* (0.23) MSI (0.23) *CRABP1* (0.21) TIL (0.21) *CDKN2A* ^b^ (0.19) *NEUROG1* (0.19) *SOCS1* (0.19)	*CACNA1G* (0.28) *MLH1* (0.26) MSI (0.26) *IGF2* (0.25) *RUNX3* (0.25) *BRAF* (0.23) *CRABP1* (0.23) *CDKN2A* ^b^ (0.21) TIL (0.21) *NEUROG1* (0.19) *SOCS1* (0.17)	*RUNX3* (0.19) *IGF2* (0.17) *NEUROG1* (0.17)

^a^Markers with degree centrality at or above the 80th percentile in the colorectal cancer network

^b^ *CDKN2A* promoter hypermethylation

*MSI* microsatellite instability, *TIL* Tumor infiltrating lymphocytes

To identify biomarkers that had higher connectivity particularly in the proximal colon cancer network compared with the distal colorectal cancer network, we calculated Cook’s distance (see Methods) for each highly-connected marker (those at or above the 80th percentile of the degree distribution in the overall colorectal cancer network) (Additional file 1: Figure S1). In Table 4, MSI showed the largest Cook’s distance (0.12) and was highly connected with other markers in the proximal colon cancer network (degree = 21), but not in the distal colorectal cancer network (degree = 9). In the proximal colon cancer network, MSI was positively correlated with *MLH1* methylation (correlation coefficient [ρ] = 0.77), *CACNA1G* methylation (ρ = 0.55), *RUNX3* methylation (ρ = 0.50), *SOCS1* methylation (ρ = 0.49), *IGF2* methylation (ρ = 0.46), *CRABP1* methylation (ρ = 0.45), *BRAF* mutation (ρ = 0.44), TIL (ρ = 0.41), *NEUROG1* methylation (ρ = 0.37), Crohn’s-like reaction (ρ = 0.37), *CDKN2A* methylation (ρ = 0.33), abundance of *Fusobacterium nucleatum* (ρ = 0.33), SIRT1 overexpression (ρ = 0.25), loss of CDX2 expression (ρ = 0.25), CCND1 overexpression (ρ = 0.25), LINE-1 methylation (ρ = 0.24), peritumoral lymphocytic reaction (ρ = 0.24), MIR155 expression (ρ = 0.22), and intratumoral periglandular reaction (ρ = 0.22). MSI was negatively correlated with *KRAS* mutation (ρ = −0.39) and loss of CDKN1A expression (ρ = −0.36), suggesting that these molecular events tended to be mutually exclusive with MSI-high. In the distal colorectal cancer network, MSI was also positively correlated with *MLH1* methylation (ρ = 0.35), TIL (ρ = 0.29), *RUNX3* methylation (ρ = 0.26), Crohn’s-like reaction (ρ = 0.24), *CACNA1G* methylation (ρ = 0.23), *SOCS1* methylation (ρ = 0.22), intratumoral periglandular reaction (ρ = 0.22), *BRAF* mutation (ρ = 0.21), and peritumoral lymphocytic reaction (ρ = 0.19). These were the subset of biomarkers positively correlated with MSI in the proximal colon cancer network, implying a partly shared mechanism by which MSI promotes perturbation of cellular and molecular functions. Moreover, the methylation markers of *MLH1*, *IGF2*, and *CACNA1G* had a higher degree in the proximal colon cancer network compared with the distal colorectal cancer network. As shown in Fig. 1, negative correlations were observed between *KRAS* mutation and other biomarkers in the proximal colon cancer network, but not in the distal colorectal cancer network. In the proximal colon cancer network, *KRAS* mutation was negatively correlated with *BRAF* mutation (ρ = −0.47), *MLH1* methylation (ρ = −0.40), MSI-high (ρ = −0.39), *RUNX3* methylation (ρ = −0.34), *CACNA1G* methylation (ρ = −0.32), *CRABP1* methylation (ρ = −0.31), *IGF2* methylation (ρ = −0.30), loss of CDX2 expression (ρ = −0.29), *SOCS1* methylation (ρ = −0.27), *CDKN2A* methylation (ρ = −0.23), *NEUROG1* methylation (ρ = −0.22), and Crohn’s-like reaction (ρ = −0.20).

**Table 4:** Markers with differential connectivity by tumor location based on Cook’s distance among highly-connected markers^a^

Highly-connected markers^a^	Degree centrality	Degree in proximal colon cancer	Degree in distal colorectal cancer	Cook’s distance
MSI	0.45	21	9	0.12
*IGF2*	0.38	18	9	0.028
*CRABP1*	0.38	16	8	0.015
*CACNA1G*	0.45	18	10	0.0071
TIL	0.43	18	10	0.0071

^a^Highly-connected markers which were at or above the 80th percentile of the degree distribution in the colorectal cancer network. The table shows markers with Cook’s distance higher than 0.005

*MSI* microsatellite instability, *TIL* tumor infiltrating lymphocytes

### Biomarker networks constructed in non-MSI-high colorectal cancer

The higher frequency of MSI-high in the proximal colon cancer network compared with the distal colorectal cancer network could potentially result in the higher connectivity of the former. Therefore, as a secondary analysis, we restricted our analysis to patients with non-MSI-high cancer, and constructed networks by tumor location. After matching by age and sex, there were 246 patients with non-MSI-high proximal colon cancer and 246 patients with non-MSI-high distal colorectal cancer (Additional file 2: Figure S2). Compared with the distal colorectal cancer network, we observed a slightly higher connectivity and more hubs in the proximal colon cancer network. The total numbers of edges was 64 in the proximal colon cancer network and 56 in the distal colorectal cancer network (Table 3). Highly connected biomarkers (with degree centrality) were *BRAF* mutation (0.19) and methylation-related markers including *CDKN2A* (0.17), *IGF2* (0.17), *RUNX3* (0.17), *CACNA1G* (0.15), *CRABP1* (0.15), and *NEUROG1* (0.15) in the proximal colon cancer network. In the distal colorectal cancer network, two biomarkers were hubs including *NEUROG1* methylation (degree centrality = 0.15) and *RUNX3* methylation (degree centrality = 0.15). Among non-MSI-high cases, *BRAF* mutation was the most distinct marker that was strongly correlated with other markers in the proximal cancer network (degree = 10), but not in the distal cancer network (degree = 3) (Cook’s distance = 0.17). In the proximal colon cancer network, *KRAS* mutation tended to be mutually exclusive only with *BRAF* mutation, indicating that the observed negative correlations of *KRAS* mutation with methylation-related markers and other biomarkers might be confounded by MSI-high in our earlier analysis.

### Biomarker networks with equal edge counts

To examine whether the difference in the number of edges across the two networks affected hub identification (designation of nodes as hubs), we modified each network by retaining 100 of the largest Spearman correlation coefficients in absolute values regardless of their *P*-values, resulting in two networks with 100 edges (Additional file 3: Figure S3). The median degree was 4.0 in the proximal colon cancer network and 1.0 in the distal colorectal cancer network, indicating that edges were confined to particular biomarkers in the proximal colon cancer network. When defined as markers with degree centrality at or above the 80th percentile in the colorectal cancer network, there were 11 hubs including methylation-related markers, MSI, *BRAF* mutation, and TIL in the proximal colon cancer network (Table 3). In contrast, only three methylation-related markers were identified as hubs in the distal colorectal cancer network. This finding supports the characteristics of the proximal colon cancer network observed in the original network analysis, where MSI and methylation-related markers were associated with perturbation of other molecular events.

### Sensitivity analyses

**Fig. 3:**
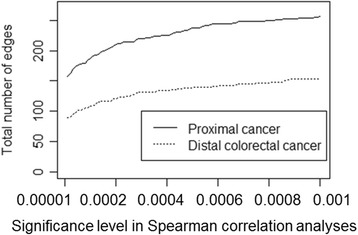
Total number of edges in biomarker networks as a function of the significance levels in Spearman correlation analyses

Across different significance levels in the Spearman correlation analyses, the total number of edges in the proximal colon cancer network was consistently greater than in the distal colorectal cancer network, supporting a more highly correlated biomarker network in proximal colon cancer (Fig. 3). In addition, we constructed biomarker networks based on the Spearman correlation analyses using binary variables for all markers and still found that the proximal colon cancer network was denser than the distal colorectal cancer network (Additional file 4: Table S1). Moreover, the proximal colon cancer remained denser when the analysis included only biomarkers with missing data in less than 20% of the patients (Additional file 4: Table S1).

## Discussion

Within a tumor molecular dataset including major mutational events, MSI, epigenetic features, protein expression status, and host immune reactions in 1380 colorectal cancer patients, we conducted a network analysis to evaluate biomarker network structure in proximal colon cancer and distal colorectal cancer. We found that in proximal colon cancer there were many highly correlated biomarkers, leading to an overall denser network as compared with distal colorectal cancer. In the distal colorectal cancer network, biomarkers had fewer connections and were less clustered, resulting in fewer hubs. This finding indicates that carcinogenic events in distal colorectal cancer tended to occur independently from each other. In the proximal colon cancer network, MSI was a hub and had differential connectivity with other biomarkers compared with the distal colorectal cancer network. In both the proximal colon and distal colorectal cancer networks, we observed common biomarkers that positively correlated with MSI including methylation-related markers, *BRAF* mutation, and immune reactions, while overexpression or loss of protein expression associated with MSI were further found only in the proximal colon cancer network. In addition to MSI, CpG island methylation in *MLH1*, *IGF2*, and *CACNA1G* genes were highly connected to other biomarkers mainly in proximal colon cancer. Among non-MSI-high patients, *BRAF* mutation status was the most distinct marker that was strongly associated with other molecular events in the proximal colon cancer network, but not in the distal colorectal cancer network. The sensitivity analyses consistently showed a denser network in proximal colon cancer when compared with the distal colorectal cancer network. Our results indicate that many molecular events occur in relation to MSI in proximal colon cancer, and that MSI and *BRAF* mutation played important roles in the carcinogenic process of proximal colon cancer.

In colorectal cancer and other types of cancer, previous biomarker network studies have shown the importance of interdependence of mutation, methylation, and protein expression [16, 17]. In a biological network, hubs (highly connected nodes) are considered to be drivers playing an essential role during tumorigenesis [14, 15]. Previous studies showed that *MLH1* promoter methylation causes mismatch repair deficiency and MSI [2, 3]. In sporadic cancer, it is not well known whether MSI causes mutations in oncogenes and tumor-suppressor genes, and aberrant protein expressions. Earlier studies reported that genomic instability conferred by somatic mutational alterations further generates mutations in oncogenes or anti-oncogenes [18, 19]. Our results suggest that the role of MSI differ by tumor anatomic location; in proximal colon cancer, MSI might lead to perturbations of many molecular events, and in distal colorectal cancer molecular events are more likely independent from MSI. Consistent with our findings, MSI and CIMP-high were highly correlated and occurred more frequently in proximal colon cancer than in distal colorectal cancer [11]. Methylation signatures measured in our study were CIMP-specific markers which were correlated with each other. Thus, higher frequencies of MSI and CIMP-high might contribute to the higher connectivity of the proximal colon cancer network. However, even when we excluded MSI cancer patients from the analyses, the higher connectivity persisted in the proximal colon cancer network. In addition, when we modified the two networks such that each had 100 edges, corresponding to the 100 most positive or negative correlation coefficients, the proximal colon cancer network remained to have more hubs and greater median degree than the distal colorectal cancer network. Our findings provide new insights into the carcinogenesis of proximal colon cancer, which possibly exhibits highly interactive biological mechanisms.

*BRAF* oncogenic mutations are observed in 10% to 20% of colorectal cancer patients and are associated with MSI-high and CIMP-high phenotypes [19, 20]. The serine/threonine-protein kinase BRAF is involved in the mitogen-activated protein kinase (MAPK) pathway, which is associated with proliferation, cell growth, and differentiation [20]. In our study, CIMP-high was associated with *BRAF* mutation in both proximal colon and distal colorectal cancer. Previous studies observed CIMP-high and *BRAF* mutations in an early-stage colorectal neoplasm, and acquisition of *BRAF* mutation was considered to be mediated by DNA hypermethylation of several genes, including *IGFBP7* and *BMP3* [18]. The close relationship between CIMP and *BRAF* mutation may drive carcinogenesis regardless of tumor location.

A large body of literature demonstrated that colorectal cancer patients with non-MSI-high and mutant *BRAF* were associated with the highest mortality, whereas patients with MSI-high/*BRAF*-wild-type showed the lowest mortality when both MSI and *BRAF* mutation status were considered [21–24]. Among proximal colon cancer patients, the non-MSI-high/*BRAF*-mutant subtype was associated with a worse prognosis [25]. Together with these previous studies, our findings suggest the critical role of *BRAF* mutation in the absence of MSI-high especially in the proximal colon carcinogenesis.

Our results showed the strong negative correlation between *BRAF* and *KRAS* mutations specifically in proximal colon cancer. Both *BRAF* and *KRAS* oncogenes encode proteins involved in the MAPK pathway, and *BRAF* mutation has been reported to be mutually exclusive with *KRAS* mutation in colorectal cancer [26, 27]. *BRAF* is more frequently mutated in the proximal colon than distal colorectum, while the frequency of *KRAS* mutation is more similar throughout the colorectum except for the cecum [11]. The biological correlation pattern between *BRAF* and *KRAS* might depend on tumor location.

The distal colorectal cancer network was less densely connected than the proximal colon cancer network. Although we did not find biomarkers with high degree centrality, the promoter methylations, *BRAF* mutation, and TIL tended to be correlated with other molecular events. Distal carcinoma was previously reported to be more responsive to drug regimens, including 5-fluorouracil-based chemotherapeutics and single-agent anti-epidermal growth factor receptor (EGFR) antibodies, while proximal carcinoma tended to show more resistance to those treatments [12, 26]. Our results from the network analysis found that oncogenic processes are relatively independent from one another in distal colorectal cancer. Taken together, our data support a biological difference between colorectal carcinoma by tumor location.

We recognize that there are several limitations to our study. Although we chose widely recognized pathological biomarkers in colorectal cancer, our data did not cover all molecular pathological markers that have been reported in the literature, such as the mutation status of *SMAD4* and *PTEN* [28, 29]. Our selection of the biomarkers might have resulted in a greater connectivity of the biomarker network in the proximal colon. Measurement errors could have existed in protein expression analyses using immunohistochemistry (IHC). Nonetheless, based on reviews by two independent pathologists, most of the IHC markers showed agreement levels generally considered to be reasonable in pathology research (kappa coefficient > 0.6). We conducted network analyses focusing on the proximal colon and distal colorectum, but not on more detailed segments (the cecum, ascending colon, hepatic flexure, transverse colon, splenic flexure, descending colon, sigmoid colon, rectosigmoid junction, and rectum). The importance of examining these segments warrants further investigations with even larger sample sizes. These results contribute to our understanding of colorectal carcinogenesis, suggesting a different mechanism by tumor location. Although our findings were corroborated by sensitivity analyses, future validation studies with independent data sets are necessary to confirm these findings.

Despite the limitations, our study has several strengths that distinguish it from previous studies. First, we measured different kinds of tumor biomarkers including somatic mutations, methylation levels, MSI status, immune reactions, and protein expressions. These measurements provided a reasonably comprehensive view of molecular interplay in the networks and enabled the identification of important carcinogenic pathways. Second, our overall sample size with 1380 cases enabled us to conduct robust network analyses using multiple biomarkers. Third, we collected colorectal tumors from hospitals and pathology laboratories across the U.S. (rather than one or a few hospitals), which increases generalizability of our findings.

## Conclusions

Biomarkers in proximal colon cancer possessed higher connectivity while those in distal colorectal cancer tended to be independent from each other. In proximal colon cancer, MSI-high and *BRAF* mutation occurred in relation to many other tumor features, indicating their important roles during carcinogenesis. Our findings highlight the necessity of a systems therapeutic approach that can target both specific and multiple pathways of the proximal colon cancer network.

## Methods

### Study population and design

Initiated in 1976, the NHS enrolled 121,701 female nurses aged 30–55 years. Established in 1986, the HPFS enrolled 51,529 male health professionals including dentists, optometrists, osteopaths, pharmacists, podiatrists, and veterinarians aged 40–75 years. Within these two cohorts, colorectal cancer patients were ascertained by biennial questionnaires, the National Death Index (for unreported lethal cases), and reviews of medical records. Study physicians reviewed medical and pathological records to retrieve detailed information on colorectal tumors including TNM stage, differentiation, and bowel subsites. For bowel subsite sections, the proximal colon included the cecum, ascending colon, hepatic flexure, and transverse colon; the distal colorectum included the splenic flexure, descending colon, sigmoid colon, rectosigmoid junction, and rectum.

We collected formalin-fixed paraffin-embedded (FFPE) archival tumor tissue blocks from pathology laboratories in U.S. hospitals where patients underwent tumor resection. A centralized pathological review of hematoxylin and eosin-stained tissue sections was conducted by a single pathologist (S.O.) for all colorectal carcinoma cases. Tumor differentiation was categorized into well to moderate or poor, based on the extent of glandular areas. Based on the availability of tumor tissue blocks, 1591 cases were selected for the molecular and pathological analyses. Patients with proximal colon cancer were matched by age and sex to patients with distal colorectal cancer by randomly selecting a patient diagnosed with proximal colon cancer from the same sex and age category (<50, 50–59, 60–69, or ≥70 years). Subsequently, out of the 1591 patients in the original dataset, 1380 patients (690 patients with proximal colon cancer and 690 patients with distal colorectal cancer) were included in the network analysis dataset. For each marker, patients were excluded from an analysis when data on that marker were not available. Percentages of patients with available biomarkers are shown in Table 1.

### Molecular pathological analysis

In the NHS and the HPFS, we utilized a colorectal cancer database which contained biomarker data on molecular alterations reported to play important roles in carcinogenic pathways in colorectal cancer. Tumor molecular and pathological markers in the database included mutational events, epigenetic features, protein expression levels, and host immune reactions in colorectal carcinoma. The analyses were performed as previously described. In FFPE tissues, DNA extraction and pyrosequencing were conducted for *BRAF* (codon 600), *KRAS* (codons 12, 13, 61, or 146), and *PIK3CA* (exons 9 and 20) [30–32]. The analysis of MSI was performed using 10 microsatellite markers (D2S123, D5S346, D17S250, BAT25, BAT26, BAT40, D18S55, D18S56, D18S67, and D18S487) [24]. We defined MSI-high as instability in ≥30% of the markers and MSI-low/microsatellite stability (MSS) as instability in <30% of the markers [24]. To quantify the amount of *Fusobacterium nucleatum* DNA, quantitative polymerase chain reaction (PCR) assay was performed using the TaqMan primer/probe sets (Applied Biosystems) for the *nusG* gene of *Fusobacterium nucleatum* and for *SLCO2A1* as a reference human gene [33]. Amplification and detection of DNA was performed with the StepOnePlus Real-Time PCR Systems (Applied Biosystems).

DNA methylation was quantified using validated bisulfite DNA treatment and real-time PCR (MethyLight) for eight CpG island specific promoters including *CACNA1G, CDKN2A* (p16), *CRABP1, IGF2, MLH1, NEUROG1, RUNX3*, and *SOCS1* [34]. The relative methylation level of long interspersed nucleotide element 1 (LINE-1) and *IGF2* differentially methylated region 0 (DMR0) were quantified by pyrosequencing [35, 36].

Immunohistochemistry (IHC) analyses were conducted for the following protein markers: AURKA [37], CCND1 [38], CD274 (PD-L1) [39], CDH1 [40], CDK8 [41], CDKN1A [42], CDKN1B [42], CDKN2A [43], CDX2 [44], nuclear CTNNB1 [45], CTSB [46], DNMT3B [47], EPAS1 [48], FASN [49], HGF [50], HIF1A [48], IGF2BP3 [51], IRS1 [52], IRS2 [53], MGMT [54], PPARG [55], PTGER2 [56], PTGS2 [6], SIRT1 [57], STAT3 [58], TP53 [59], VDR [60], cytoplasmic YAP1 [61], and JC virus T-antigen (JCVT) [62]. For T cells in tumor tissue, we conducted IHC and image analysis on tissue microarray to measure densities of CD3+, CD8+, CD45RO+, and FOXP3^+^ cells (cells/mm^2^) [63]. Lymphocytic reactions were examined by the pathologist (S.O.) for tumor-infiltrating lymphocytes (i.e., lymphocytes on top of neoplastic epithelial cells, TIL), peritumoral lymphocytic reaction, intratumoral periglandular reaction, and Crohn’s-like reaction [64].

RNA was extracted from the colorectal tumor tissue and adjacent non-tumor tissue within FFPE samples, and cDNA was synthesized as previously described [4]. The expression levels of *MIR21* and *MIR155* were quantified using miScript PCR System (Qiagen, Valencia, CA) with the StepOnePlus Real-Time PCR Systems (Applied Biosystems, San Diego, CA) [65].

### Statistical and network analysis

To compare demographic characteristics of proximal colon cancer patients with distal colorectal cancer patients, a chi-squared test was used to compare two or more categorical variables by location, and a t-test was conducted to compare age by location.

In the correlation network analysis [66], a node represented a tumor tissue biomarker. The structure of correlations was captured by placing an edge between any two nodes whose associated biomarkers exhibited statistically significant Spearman correlation, where the significance level for correlations was chosen to be 3.5 × 10^−5^ (= 0.05/1431, based on the Bonferroni correction). The pairwise correlation analysis was conducted as a complete case analysis in which patients without either biomarker information were excluded from the analysis. In the correlation analyses, we used continuous (for MSI and markers measuring levels of methylation, T-cell densities, miRNA expression, and *Fusobacterium nucleatum*), ordinal (for markers measuring tumor protein expression levels), and binary (for markers assessing mutation status) variables. The degree of a node was defined as the number of edges adjacent to the given node. We used a Kolmogorov-Smirnov (K-S) test to evaluate the distance between the cumulative degree distributions of the proximal colon cancer network and the distal colorectal cancer network. For each marker, degree centrality was computed as the fraction of nodes to which a node was connected (i.e., degree of a node divided by the number of all nodes in the network). A network node (marker) showing a high degree of connectedness, often referred to as a hub, is more likely to play an essential role in the disease network [14]. In the current study, hubs were defined as nodes with degree centrality greater than the 80th percentile based on overall colorectal cancer network that pooled both proximal colon and distal colorectal cancer. In each network, average clustering coefficient quantifies the overall clustering of nodes, indicating the tendency of markers to clustering together in the network [67]. For each node, a clustering coefficient was computed as the proportion of directly connected neighbors, and then an average of clustering coefficients was calculated in the network. To identify the markers that show distinct correlation patterns in proximal colon cancer versus distal colorectal cancer, we computed Cook’s distance based on linear regression analysis [68]. In this analysis, the degree of each node in the distal colorectal cancer networks was regressed on the degree of each node in the proximal colon cancer networks.

To assess the robustness of our results from the network analysis, we conducted permutation tests and sensitivity analyses. First, to evaluate the null hypothesis of no difference in network edge counts by tumor location, we permutated the dataset 10,000 times by randomly assigning tumor location in 1380 patients. Each time, networks were constructed by tumor location, and the difference in the total number of edges was computed. The two-sided *P* value was obtained as the proportion of random permutations that resulted in a difference in edge counts that was greater than or equal to the observed difference. Similarly, another permutation test was conducted by permuting values of each biomarker in 690 patients with proximal colon cancer and in 690 patients with distal colorectal cancer separately. In this process, we broke the linkage between each pair of biomarkers but retained imbalanced data (e.g., difference in mutation frequency) between tumor location. Second, across different significance levels of the Spearman correlation, we compared the number of edges in the proximal colon cancer network with that in the distal colorectal cancer network. Third, we used binary variables for all markers in the Spearman correlation analysis, and we constructed network models by tumor location. Fourth, we conducted a sensitivity analysis to include only selected markers that were available for more than 80% of the patients (*BRAF*, *KRAS*, *PIK3CA*, *MLH1* methylation, *CDKN2A* methylation, *CACNA1G* methylation, *CRABP1* methylation, *IGF2* methylation, *NEUROG1* methylation, *RUNX3* methylation, *SOCS1* methylation, MSI, LINE-1 methylation level, PTGS2, peritumoral lymphocytic reaction, intratumoral periglandular reaction, and TILs).

All the statistical analyses were carried out with SAS software (version 9.4, SAS Institute, Cary, NC). Network analysis was carried out with Python (version 2.7). All *P* values were two-sided.

## Additional files

Additional file 1: Figure S1. The biomarker network in colorectal cancer. A node represents a molecular feature, and an edge specifies the Spearman correlation between two markers with a significance level of 3.5 × 10^−5^ (0.05/1431, based on the Bonferroni correction). The red line indicates a positive correlation, and the blue line indicates a negative correlation. The line width is proportional to a correlation coefficient. CDKN2A (IHC), protein expression of CDKN2A; CDKN2A, methylation level of *CDKN2A*; LINE-1, methylation level of long interspersed nucleotide element 1; MSI, microsatellite instability; TIL, lymphocytes on top of neoplastic epithelial cells. (TIFF 344 KB)

Additional file 2: Figure S2. The biomarker networks in non-MSI-high colorectal cancer; proximal colon cancer network (A), and distal colorectal cancer network (B). (TIFF 325 KB)

Additional file 3: Figure S3. The biomarker networks with the same number of edges; proximal colon cancer network (A), and distal colorectal cancer network (B). (TIFF 311 KB)

Additional file 4: Table S1. Network characteristics by tumor location. (DOCX 16.4 KB)

## Abbreviations

CIMP: CpG island methylator phenotype
FFPE: Formalin-fixed paraffin-embedded
HPFS: Health Professionals Follow-up Study
IGF2 DMR0: *IGF2* differentially methylated region 0
IHC: Immunohistochemistry
JCVT: JC Virus T-Antigen
K-S test: Kolmogorov-Smirnov test
MAPK: Mitogen-activated protein kinase
MSI: Microsatellite instability
MSS: Microsatellite instability-low/microsatellite stability
NHS: Nurses’ Health Study
PCR: Quantitative polymerase chain reaction
SD: Standard deviation
TIL: Tumor infiltrating lymphocytes
