# Supplementary figures and images for: Biomarker correlation network in colorectal carcinoma by tumor anatomic location
Source: BMC Bioinformatics. 2017 Jun 17;18:304. doi: 10.1186/s12859-017-1718-5 (PMC5474023; doi:10.1186/s12859-017-1718-5)

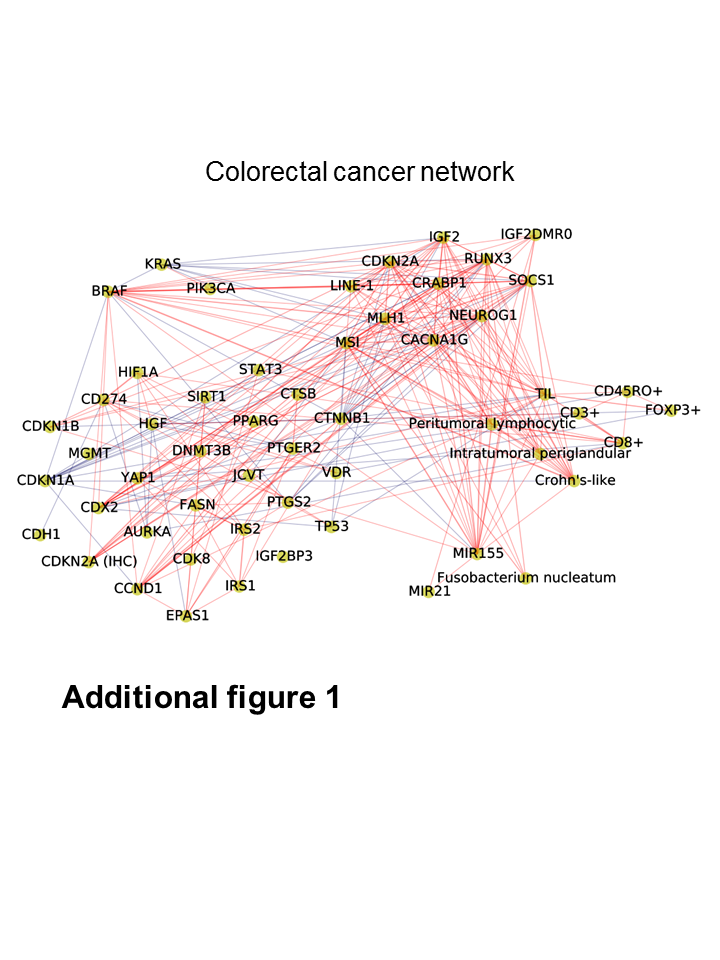

Supplement: Supplementary file 1 — The biomarker network in colorectal cancer. A node represents a molecular feature, and an edge specifies the Spearman correlation between two markers with a significance level of 3.5 × 10^−5^ (0.05/1431, based on the Bonferroni correction). The red line indicates a positive correlation, and the blue line indicates a negative correlation. The line width is proportional to a correlation coefficient. CDKN2A (IHC), protein expression of CDKN2A; CDKN2A, methylation level of CDKN2A; LINE-1, methylation level of long interspersed nucleotide element 1; MSI, microsatellite instability; TIL, lymphocytes on top of neoplastic epithelial cells. (TIFF 344 KB) [file 12859_2017_1718_MOESM1_ESM.tif]

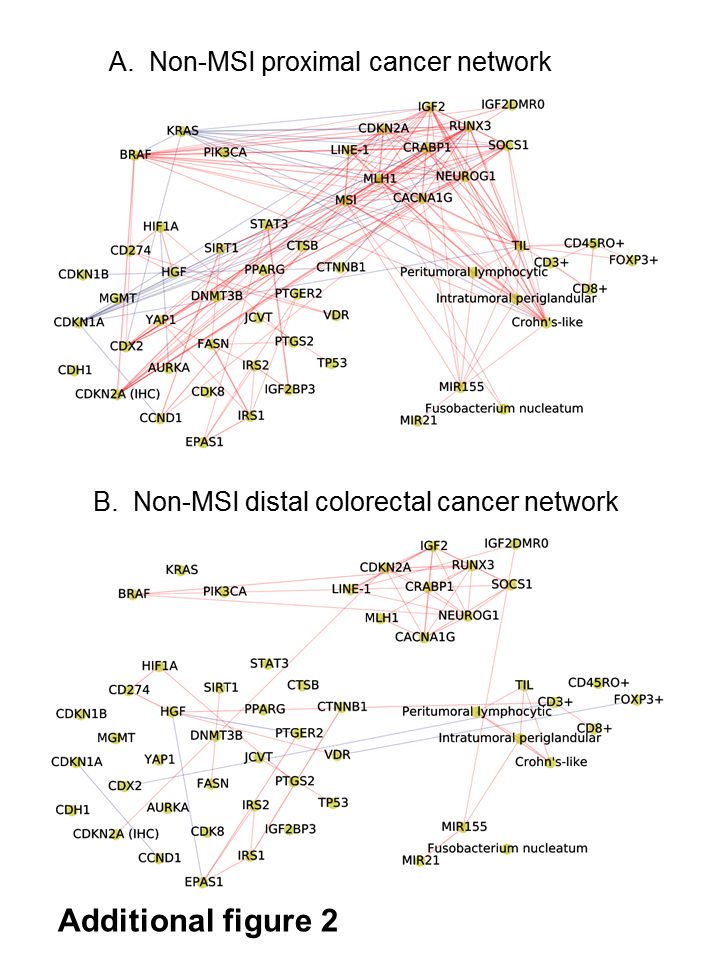

Supplement: Supplementary file 2 — The biomarker networks in non-MSI-high colorectal cancer; proximal colon cancer network (A), and distal colorectal cancer network (B). (TIFF 325 KB) [file 12859_2017_1718_MOESM2_ESM.tif]

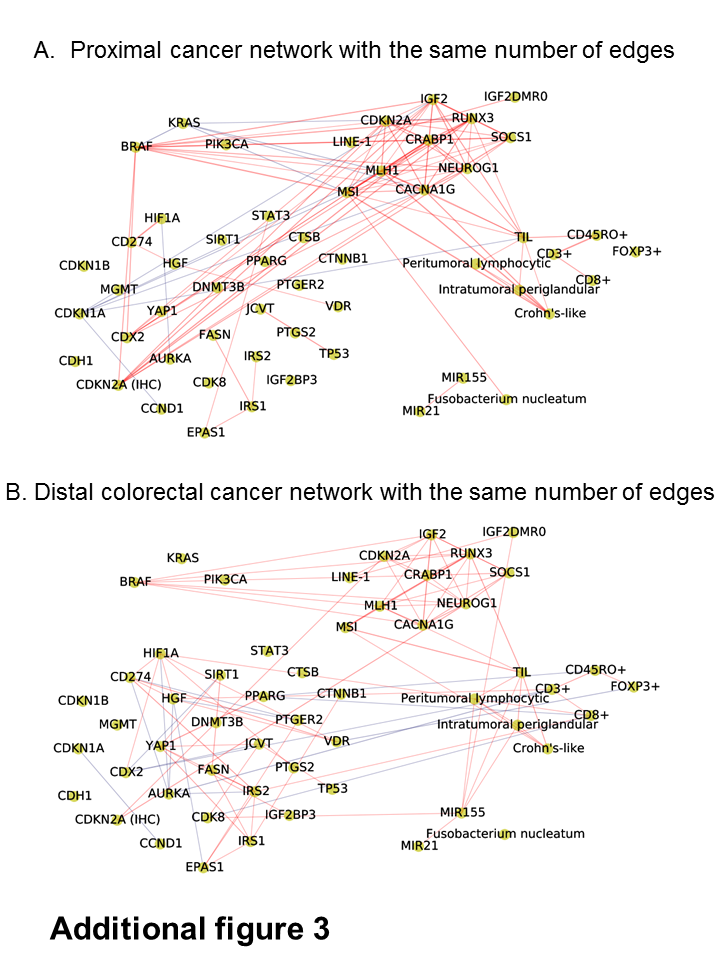

Supplement: Supplementary file 3 — The biomarker networks with the same number of edges; proximal colon cancer network (A), and distal colorectal cancer network (B). (TIFF 311 KB) [file 12859_2017_1718_MOESM3_ESM.tif]
