# Supplementary material for: Biomarker correlation network in colorectal carcinoma by tumor anatomic location
Source: BMC Bioinformatics. 2017 Jun 17;18:304. doi: 10.1186/s12859-017-1718-5 (PMC5474023; doi:10.1186/s12859-017-1718-5)
Supplement: Supplementary file 4 — Network characteristics by tumor location. (DOCX 16.4 KB) [file 12859_2017_1718_MOESM4_ESM.docx]

**Additional table 1. Network characteristics by tumor location**

|  |  | Total (N=1,380) | Proximal colon cancer (N=690) | Distal colorectal cancer (N=690) |
| --- | --- | --- | --- | --- |
| Correlation network based on binary variables | | |  |  |
|  | Number of nodes | 54 | 54 | 54 |
|  | Median degree | 3.5 | 2.0 | 1.0 |
|  | Number of edges | 200 | 140 | 90 |
|  | Average clustering coefficient | 0.46 | 0.51 | 0.32 |
|  | Hubs^a^ (degree centrality) | MSI (0.45)  *MLH1* (0.43)  *CACNA1G* (0.38)  *IGF2* (0.38) *SOCS1* (0.38) *CRABP1* (0.36)  *RUNX3* (0.36)  *NEUROG1* (0.34)  TIL (0.34)  *BRAF* (0.30) *CDKN1A* (0.26) Intratumoral periglandular reaction (0.26) | MSI (0.38)  *MLH1* (0.36) *CRABP1* (0.32) *IGF2* (0.32)  *RUNX3* (0.32) *CACNA1G* (0.30)  TIL (0.28) | *CACNA1G* (0.26) |
| Correlation network based on selected biomarkers^b^ | | |  |  |
|  | Number of nodes | 17 | 17 | 17 |
|  | Median degree | 13.0 | 12.0 | 8.0 |
|  | Number of edges | 97 | 84 | 55 |
|  | Average clustering coefficient | 0.83 | 0.80 | 0.69 |
|  | Hubs^a^ (degree centrality) | *RUNX3* (0.94)  *CACNA1G* (0.88)  *CRABP1* (0.88)  *IGF2* (0.88)  *MLH1* (0.88)  MSI (0.88) | *MLH1* (0.88)  MSI (0.88) | - |

^a^Markers with degree centrality at or above the 80th percentile in the colorectal cancer network.

^b^Selected markers include those available in more than 80% of the cases.

MSI, microsatellite instability; TIL, tumor infiltrating lymphocytes.
